# Human Biodistribution and Radiation Dosimetry of the P-Glycoprotein Radiotracer [^11^C]Metoclopramide

**DOI:** 10.1007/s11307-021-01582-4

**Published:** 2021-01-22

**Authors:** Martin Bauer, Sandra Barna, Matthias Blaickner, Konstantin Prosenz, Karsten Bamminger, Verena Pichler, Nicolas Tournier, Marcus Hacker, Markus Zeitlinger, Georgios Karanikas, Oliver Langer

**Affiliations:** 1 Department of Clinical Pharmacology, Medical University of Vienna, Vienna, Austria; 2 Preclinical Molecular Imaging, AIT Austrian Institute of Technology GmbH, Seibersdorf, Austria; 3 Center for Medical Physics Biomedical Engineering, Medical University of Vienna, Vienna, Austria; 4 Department of Biomedical Imaging und Image-guided Therapy, Division of Nuclear Medicine, Medical University of Vienna, Vienna, Austria; 5 Laboratoire d’Imagerie Biomédicale Multimodale (BioMaps), CEA, CNRS, Inserm, Service Hospitalier Frédéric Joliot, Université Paris-Saclay, Orsay, France

**Keywords:** [^11^C]metoclopramide, PET, Biodistribution, Dosimetry, P-glycoprotein

## Abstract

**Purpose:**

To assess in healthy volunteers the whole-body distribution and dosimetry of [^11^C]metoclopramide, a new positron emission tomography (PET) tracer to measure P-glycoprotein activity at the blood-brain barrier.

**Procedures:**

Ten healthy volunteers (five women, five men) were intravenously injected with 387 ± 49 MBq of [^11^C]metoclopramide after low dose CT scans and were then imaged by whole-body PET scans from head to upper thigh over approximately 70 min. Ten source organs (brain, thyroid gland, right lung, myocardium, liver, gall bladder, left kidney, red bone marrow, muscle and the contents of the urinary bladder) were manually delineated on whole-body images. Absorbed doses were calculated with QDOSE (ABX-CRO) using the integrated IDAC-Dose 2.1 module.

**Results:**

The majority of the administered dose of [^11^C]metoclopramide was taken up into the liver followed by urinary excretion and, to a smaller extent, biliary excretion of radioactivity. The mean effective dose of [^11^C]metoclopramide was 1.69 ± 0.26 μSv/MBq for female subjects and 1.55 ± 0.07 μSv/MBq for male subjects. The two organs receiving the highest radiation doses were the urinary bladder (10.81 ± 0.23 μGy/MBq and 8.78 ± 0.89 μGy/MBq) and the liver (6.80 ± 0.78 μGy/MBq and 4.91 ± 0.74 μGy/MBq) for female and male subjects, respectively.

**Conclusions:**

[^11^C]Metoclopramide showed predominantly renal excretion, and is safe and well tolerated in healthy adults. The effective dose of [^11^C]metoclopramide was comparable to other ^11^C-labeled PET tracers.

**Supplementary Information:**

The online version contains supplementary material available at 10.1007/s11307-021-01582-4.

## Introduction

P-glycoprotein (P-gp, encoded by the *ABCB1* gene) is a membrane transporter which accepts a variety of endogenous substances and drugs as its substrates [1]. It is expressed in the luminal membranes of the blood brain-barrier (BBB) and the small intestine and in the apical membranes of hepatocytes and kidney proximal tubule cells. P-gp plays an important role in limiting the brain entry of various drugs, and in the intestinal absorption and biliary and urinary excretion of drugs [1]. The activity and/or abundance of P-gp may vary among individuals due to genetic polymorphisms, disease states and age. Furthermore, drug intake may modulate the activity of P-gp potentially leading to drug-drug interactions and adverse effects [1].

Positron emission tomography (PET) with radiolabeled P-gp substrates, such as racemic [^11^C]verapamil [2, 3], (*R*)-[^11^C]verapamil [4, 5] and [^11^C]*N*-desmethyl-loperamide [6], has been proposed as a useful tool to measure P-gp activity at the BBB in health and disease. However, these radiotracers are very efficiently transported by P-gp at the BBB (“avid” P-gp substrates) leading to very low brain uptake and limited sensitivity to measure disease-induced alterations in P-gp activity at the BBB [7]. [^11^C]Metoclopramide is a weak P-gp substrate, which shows higher baseline brain uptake than previously described “avid” P-gp substrates and may thus possess better sensitivity to measure P-gp activity at the BBB, in particular in conditions in which the activity of P-gp is upregulated (*e.g.* drug-resistant epilepsy) [8–12].

The aim of this study was to assess the whole-body biodistribution of [^11^C]metoclopramide in female and male healthy volunteers and to calculate the organ dosimetry and total body effective dose.

## Materials and Methods

The study protocol was approved by the Medical University of Vienna Institutional Ethics committee, registered under EudraCT 2017–000989-30 and written informed consent was obtained from all subjects before enrolment. 10 healthy volunteers were included into the study, 5 men (mean age: 36 ± 12 years, mean weight: 79 ± 9 kg) and 5 women (mean age: 30 ± 9 years, mean weight: 66 ± 8 kg). Pre-study evaluation included medical history and a physical examination with vital signs, laboratory tests, electrocardiogram, routine blood and urine laboratory assessments and urine drug screening. All subjects were free of any medication for at least two weeks before the imaging session.

### Radiotracer Synthesis

[^11^C]Metoclopramide was synthesized by *O*-[^11^C]methylation of *O*-desmethyl-metoclopramide (GMP-grade, ABX advanced biochemical compounds, Radeberg, Germany) as described previously [13] and formulated in sterile phosphate-buffered saline solution containing 8.6 % (*v*/v) ethanol. Molar activity at the time of radiotracer injection was 242 ± 135 GBq/μmol and radiochemical purity was greater than 98 %.

### Radiotracer Administration and PET/CT Protocol

Subjects were placed in supine position on a Biograph TruePoint 64 PET/CT scanner (Siemens Healthcare USA). A low-dose CT scan was performed for attenuation correction purposes followed by administration of [^11^C]metoclopramide over approximately 20 s as an intravenous (i.v.) bolus (387 ± 49 MBq, corresponding to 2.3 ± 1.5 nmol or 0.7 ± 0.5 μg of unlabeled metoclopramide). Then, four consecutive static whole-body PET acquisitions, which each covered 6–7 overlapping bed positions (skull to mid-tight) with an individual frame length of 1, 2, 3 and 5 min, respectively, were conducted. After the imaging session subjects were asked to empty their urinary bladders and aliquots of urine were measured for radioactivity in a Packard Cobra II auto-gamma counter (Packard Instrument Company, Meriden, Connecticut, USA). Decay-corrected urinary radioactivity concentrations were multiplied by the collected urine volume to obtain the amount of radioactivity excreted into urine.

### Image Analysis and Dosimetry Calculations

Attenuation-corrected PET images were analyzed using PMOD 3.6 (PMOD Technologies Ltd., Zürich, Switzerland). The volumes of interest (VOI) were drawn on each respective PET/CT image, providing 4 VOIs for each organ corresponding to the 4 imaging time points. Organs and tissues selected were brain, thyroid gland, right lung, myocardium, liver, gall bladder, left kidney, urinary bladder, red bone marrow (L3 to L5) and muscle (VOI from the *quatriceps femoris* corresponding to a volume of approximately 100 cm^3^). Absorbed dose was calculated using the MIRD (Medical Internal Radiation Dose) value methodology [14] with QDOSE software (ABX-CRO advanced pharmaceutical services Forschungsgesellschaft mbH), version 1.1.3, more specifically with the integrated IDAC-Dose 2.1 module which bases the organs’ masses on the International Commission on Radiological Protection (ICRP) reference phantom [15]. The time-integrated activity in the source regions was calculated by linear interpolation of the very short time span between radiotracer injection and the first PET/CT acquisition, applying the trapezoidal rule to integrate from the first to the last measured time point and assuming radioactive decay only after the last time point. The resulting time-integrated activity coefficients (formerly termed “residence times”) served as input for the dose calculations with IDAC-Dose 2.1, assuming no other form of excretion for the remainder (*i.e.* the total number of disintegrations in the body minus the disintegrations in the sampled source organs). Activity concentration in the non-segmented parts of lung and kidney were assumed to be identical with the segmented parts. Radioactivity concentrations in available urine samples were incorporated into the dosimetry calculations by assigning the time point of urine collection as fifth time point for the urinary bladder content. For display purposes, time-activity curves were expressed in units of standardized uptake value (SUV = (radioactivity per g/injected radioactivity) x body weight). All values are stated as mean ± standard deviation (SD).

## Results

**Fig. 1.**
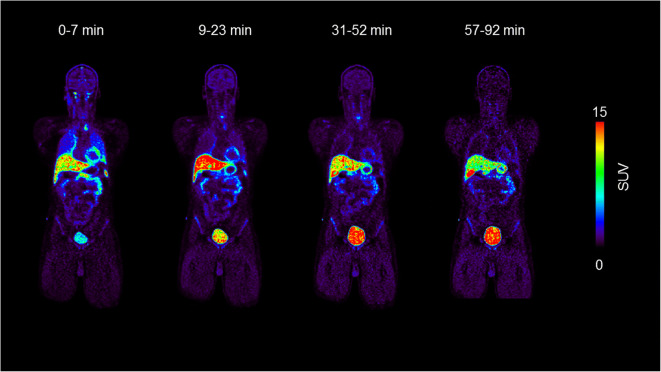
Coronal whole-body views showing biodistribution in one representative male subject (34 years, 92 kg) at different time points after injection of 353 MBq [^11^C]metoclopramide. Radiation scale is given in SUV units and set from 0 to 15.

**Fig. 2.**
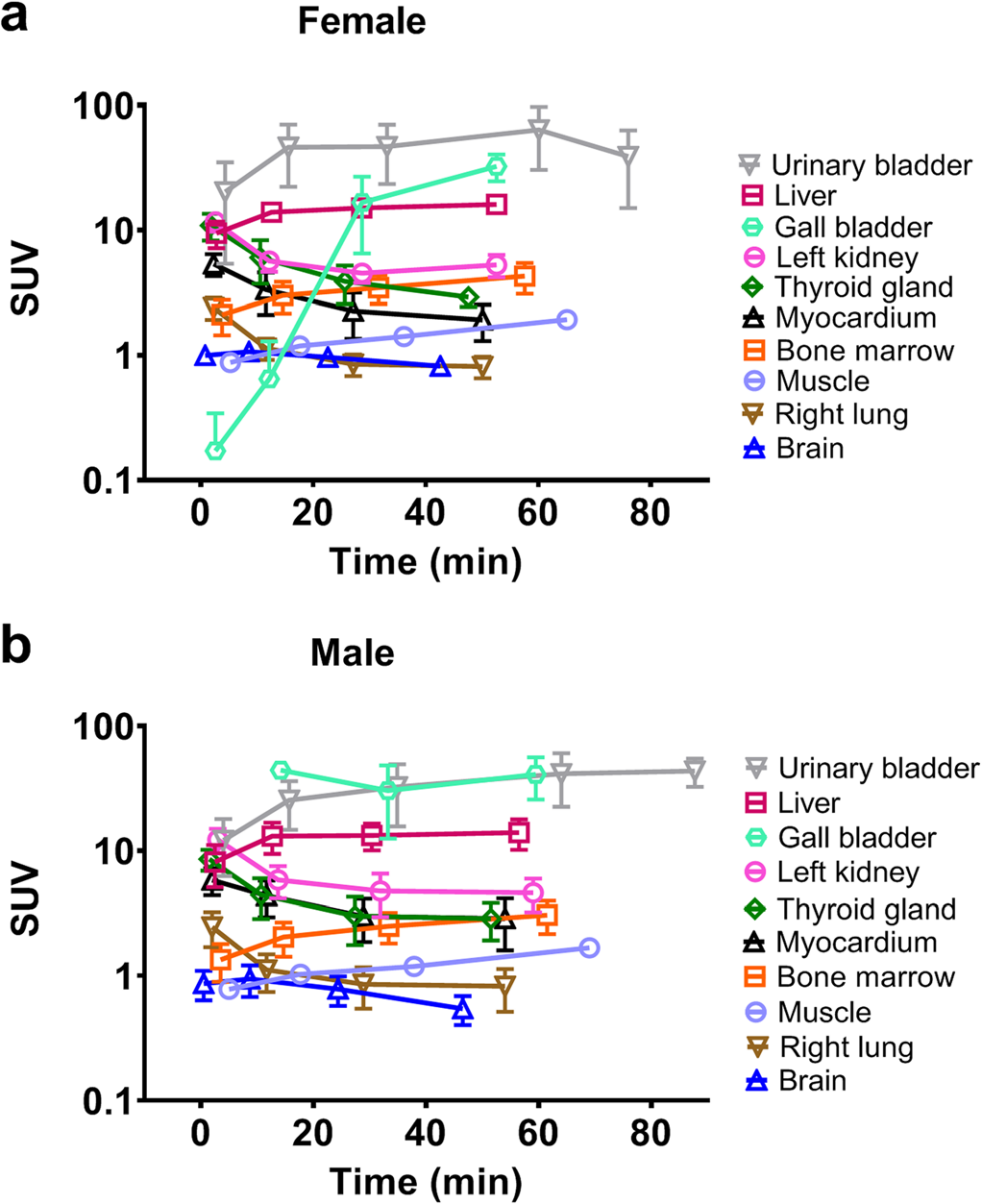
Mean (± SD) time-activity curves for [^11^C]metoclopramide in different organs for female (a, *n* = 5) and male (b, *n* = 5) subjects. The last time point of the urinary bladder curve represents the sampled urine value.

PET/CT scans of 10 healthy volunteers (5 female and 5 male subjects) were fully analyzed. The first whole-body PET/CT acquisition was started immediately after radiotracer injection followed by acquisitions at mean starting time points of 8 (range: 7–9 min), 22 (range: 20–31 min) and 42 min (range: 38–57 min) after injection. Whole-body PET images at the four investigated time points are shown for one representative male subject in Fig. 1. The liver and the urinary bladder were the organs with visually highest radioactivity uptake. At later time points, the gall bladder was also visible on the PET images. In 3 female and 2 male subjects, urine was sampled at the end of the last PET acquisition (*i.e.* at 74–101 min after radiotracer injection) and counted for radioactivity. In these subjects, the average amount of radioactivity excreted into the urine corresponded to 12 ± 3 % of the injected dose (corrected for radioactive decay). Time-activity curves in female and male subjects for all analyzed organs are shown in Fig. 2. SUV values were highest in the urinary bladder, liver and gall bladder and lowest in the brain, muscle and lungs. The SUV values from the urine samples collected at the end of the PET scan were in good agreement with the PET-measured urinary bladder curves. The sampled organs’ contribution to the total residence time was approximately 25 %, with the rest being attributed to the remainder. In Table 1, the 10 highest, calculated absorbed organ doses are presented. The urinary bladder and the liver received the highest absorbed doses. Total effective doses were 4.19 ± 0.08 μSv/MBq and 4.16 ± 0.08 μSv/MBq for female and male subjects, respectively, which corresponded for the administered radioactivity amount of approximately 400 MBq to 1.69 ± 0.26 mSv and 1.55 ± 0.07 mSv, respectively. When comparing the group in which the excreted urine measurement was considered in the dosimetry calculation and the group in which no urine was sampled after the PET/CT scan, absorbed urinary bladder wall doses were only slightly higher (11 %) for the former group and effective doses were similar for both groups (2 % difference).

**Table 1.** Absorbed organ doses and effective dose according to ICRP publication 103 [21] resulting from [^11^C]metoclopramide

	**Female**		**Male**	
**Organ**	**Absorbed dose**	**Absorbed dose**	**Absorbed dose**	**Absorbed dose**
	**[μGy/MBq]**	**[mGy]**	**[μGy/MBq]**	**[mGy]**
**Adrenals**	5.17 ± 0.16	2.08 ± 0.30	4.82 ± 0.11	1.79 ± 0.07
**Gall bladder wall**	5.65 ± 0.32	2.27 ± 0.34	5.38 ± 0.65	1.99 ± 0.20
**Kidneys**	4.72 ± 0.48	1.89 ± 0.32	4.18 ± 0.70	1.55 ± 0.21
**Liver**	6.80 ± 0.78	2.74 ± 0.53	4.91 ± 0.74	1.82 ± 0.25
**Lymphatic nodes**	4.57 ± 0.39	1.64 ± 0.27	4.58 ± 0.04	1.70 ± 0.09
**Pancreas**	4.86 ± 0.97	1.95 ± 0.28	5.01 ± 0.66	1.86 ± 0.07
**Rectosigmoid colon wall**	4.84 ± 0.30	1.95 ± 0.34	4.30 ± 0.10	1.60 ± 0.10
**Thymus**	4.20 ± 0.11	1.68 ± 0.24	4.35 ± 0.87	1.62 ± 0.09
**Urethers**	5.14 ± 0.69	2.07 ± 0.31	5.19 ± 0.45	1.93 ± 0.10
**Urinary bladder wall**	10.81 ± 0.23	4.40 ± 1.26	8.78 ± 0.89	3.26 ± 0.36
**Effective Dose**	**[**μSv**/MBq]**	**[mSv]**	**[μSv/MBq]**	**[mSv]**
	4.19 ± 0.08	1.69 ± 0.26	4.16 ± 0.08	1.55 ± 0.07

## Discussion

The aim of this study was to measure the biodistribution and organ dosimetry of [^11^C]metoclopramide, a new PET tracer for assessing the activity of P-gp at the BBB [8–12]. [^11^C]Metoclopramide is structurally identical with metoclopramide, a clinically approved antiemetic and gastroprokinetic drug, which is administered either orally or i.v. at a clinical dose of 10 mg. Previous studies have shown that following oral or i.v. administration, metoclopramide is metabolized in the liver, followed by excretion of its metabolites into the urine [16]. After administration of a single i.v. therapeutic dose of metoclopramide (10 mg) to healthy volunteers, approximately 50 % of the administered dose was excreted into the urine within 36 h after administration, whereof approximately one third was in the form of unmetabolized parent metoclopramide, as reported in the literature [16].

In the present study, we investigated the whole-body distribution of a microdose (0.7 ± 0.5 μg) of i.v. administered [^11^C]metoclopramide with PET/CT. [^11^C]Metoclopramide PET/CT was well tolerated in all subjects without the occurrence of adverse events. In good agreement with previous studies, we found that the majority of the administered radioactivity was taken up into the liver with approximately 12 % of the injected dose being excreted into the urine over the short time duration of the PET examination (approximately 70 min). The major radiolabeled metabolite of [^11^C]metoclopramide in human plasma was identified in our previously published study as the corresponding ^11^C-labeled *N*-O-glucuronide [11]. As it has been shown that the *N*-O-glucuronide was also a major metabolite of metoclopramide in the urine [17], it can be assumed that part of the radioactivity excreted into urine was in the form of the ^11^C-labeled *N*-O-glucuronide of [^11^C]metoclopramide. The visibility of the gall bladder and duodenum on the PET images indicated that [^11^C]metoclopramide also underwent, to a lower extent, biliary excretion [18].

In agreement with the whole-body distribution data (Fig. 1), the highest dose was received by the urinary bladder, followed by the liver and the gall bladder (Table 1). The mean effective dose of [^11^C]metoclopramide was 4.19 ± 0.08 μSv/MBq for female and 4.16 ± 0.08 μSv/MBq for male subjects, which was in the typical dosimetry range of other ^11^C-labeled PET tracers (mean 5.9 μSv/MBq, range 3.2–8.9 μSv/MBq) [19].

PET imaging is increasingly used to investigate transporter activities at other biological barriers than the BBB [20]. Knowledge of the whole-body distribution of [^11^C]metoclopramide in humans may thus pave the way for measuring P-gp activity in other organs than the brain.

## Conclusion

Following i.v. injection of a microdose of [^11^C]metoclopramide in healthy volunteers, the majority of administered radioactivity was taken up by the liver followed by urinary excretion, which is in good agreement with previously published data on the excretion of metoclopramide at therapeutic doses. [^11^C]Metoclopramide PET/CT was safe and well tolerated in healthy adults and mean effective doses were comparable to other ^11^C-labeled PET tracers, enabling the future clinical use of [^11^C]metoclopramide as a PET tracer for P-gp activity.

## Supplementary Information

ESM 1 (PDF 140 kb)
