# Supplementary material for: Human Biodistribution and Radiation Dosimetry of the P-Glycoprotein Radiotracer [11C]Metoclopramide
Source: Mol Imaging Biol. 2021 Jan 22;23(2):180–5. doi: 10.1007/s11307-021-01582-4 (PMC7910245; doi:10.1007/s11307-021-01582-4)
Supplement: Supplementary file 1 — (PDF 140 kb) [file 11307_2021_1582_MOESM1_ESM.pdf]

P1		
Female Adult Phantom		[MBq]
07.02.2019	14:35:34	290.47
**Organ**	**[mGy/MBq]**	**[mGy]**
Adipose	4.24E-03	1.23E+00
Adrenals	5.16E-03	1.50E+00
Alveolar-inter	3.60E-03	1.05E+00
Brain	9.92E-04	2.88E-01
Breasts	4.23E-03	1.23E+00
Bronchial bou	4.28E-03	1.24E+00
Bronchial seq	4.26E-03	1.24E+00
Bronchiolar s	3.56E-03	1.03E+00
Colon	3.97E-03	1.15E+00
Cortical bone	2.80E-03	8.15E-01
ET region	2.38E-03	6.91E-01
ET1 surface	2.16E-03	6.28E-01
ET2 surface	2.38E-03	6.91E-01
Extrathoracic	4.46E-03	1.30E+00
Eye lenses	3.06E-03	8.89E-01
Gallbladder w	5.60E-03	1.63E+00
Heart wall	2.27E-03	6.61E-01
Kidneys	4.81E-03	1.40E+00
Left colon wa	3.85E-03	1.12E+00
Liver	6.22E-03	1.81E+00
Lungs	3.81E-03	1.11E+00
Lymphatic no	4.61E-03	1.34E+00
Muscle	1.36E-03	3.94E-01
Oesophagus w	3.80E-03	1.10E+00
Oral mucosa	3.97E-03	1.15E+00
Ovaries	5.58E-03	1.62E+00
Pancreas	4.91E-03	1.43E+00
Pituitary glan	3.60E-03	1.05E+00
Prostate	0.00E+00	0.00E+00
Rectosigmoid	4.61E-03	1.34E+00
Red marrow	3.10E-03	9.00E-01
Right colon w	3.77E-03	1.09E+00
SI wall	4.09E-03	1.19E+00
Salivary gland	4.06E-03	1.18E+00
Skin	3.44E-03	9.98E-01
Spleen	3.90E-03	1.13E+00
Stomach wall	3.75E-03	1.09E+00
Systemic lym	4.63E-03	1.35E+00
Testes	0.00E+00	0.00E+00
Thoracic lymp	4.48E-03	1.30E+00
Thymus	4.28E-03	1.24E+00
Thyroid	3.60E-03	1.05E+00
Tongue	2.22E-03	6.46E-01
Tonsils	4.00E-03	1.16E+00
Ureters	5.12E-03	1.49E+00

P2		
Male Adult Phantom		[MBq]
21.02.2019	14:40:58	367.04
**Organ**	**[mGy/MBq]**	**[mGy]**
Adipose	4.25E-03	1.56E+00
Adrenals	4.72E-03	1.73E+00
Alveolar-inter	3.50E-03	1.28E+00
Brain	9.97E-04	3.66E-01
Breasts	4.08E-03	1.50E+00
Bronchial bou	4.12E-03	1.51E+00
Bronchial seq	4.10E-03	1.51E+00
Bronchiolar s	3.59E-03	1.32E+00
Colon	3.91E-03	1.44E+00
Cortical bone	2.98E-03	1.10E+00
ET region	2.11E-03	7.75E-01
ET1 surface	1.81E-03	6.63E-01
ET2 surface	2.11E-03	7.75E-01
Extrathoracic	4.24E-03	1.56E+00
Eye lenses	3.25E-03	1.19E+00
Gallbladder w	4.99E-03	1.83E+00
Heart wall	2.28E-03	8.36E-01
Kidneys	3.51E-03	1.29E+00
Left colon wa	3.77E-03	1.38E+00
Liver	4.43E-03	1.62E+00
Lungs	3.73E-03	1.37E+00
Lymphatic no	4.60E-03	1.69E+00
Muscle	1.11E-03	4.07E-01
Oesophagus w	3.91E-03	1.43E+00
Oral mucosa	4.14E-03	1.52E+00
Ovaries	0.00E+00	0.00E+00
Pancreas	4.97E-03	1.82E+00
Pituitary glan	3.48E-03	1.28E+00
Prostate	5.88E-03	2.16E+00
Rectosigmoid	4.33E-03	1.59E+00
Red marrow	3.21E-03	1.18E+00
Right colon w	3.86E-03	1.41E+00
SI wall	3.97E-03	1.46E+00
Salivary gland	3.90E-03	1.43E+00
Skin	3.44E-03	1.26E+00
Spleen	3.85E-03	1.41E+00
Stomach wall	3.66E-03	1.34E+00
Systemic lym	4.65E-03	1.71E+00
Testes	4.34E-03	1.59E+00
Thoracic lymp	4.46E-03	1.64E+00
Thymus	4.37E-03	1.60E+00
Thyroid	2.92E-03	1.07E+00
Tongue	2.50E-03	9.18E-01
Tonsils	4.03E-03	1.48E+00
Ureters	5.22E-03	1.92E+00

Urinary bladc	8.80E-03	2.56E+00
Uterus/Cervi›	6.20E-03	1.80E+00
	**[mSv/MBq]**	**[mSv]**
ED ICRP 103	4.10E-03	1.19E+00
ED ICRP 60	4.34E-03	1.26E+00

Urinary bladc	8.62E-03	3.16E+00
Uterus/Cervi›	0.00E+00	0.00E+00
	**[mSv/MBq]**	**[mSv]**
ED ICRP 103	4.05E-03	1.49E+00
ED ICRP 60	3.93E-03	1.44E+00

P3		
Male Adult Phantom		[MBq]
07.03.2019	14:24:37	397.65
**Organ**	**[mGy/MBq]**	**[mGy]**
Adipose	4.27E-03	1.70E+00
Adrenals	4.68E-03	1.86E+00
Alveolar-inte	3.29E-03	1.31E+00
Brain	1.07E-03	4.26E-01
Breasts	4.08E-03	1.62E+00
Bronchial bou	4.10E-03	1.63E+00
Bronchial seq	4.08E-03	1.62E+00
Bronchiolar s	3.39E-03	1.35E+00
Colon	3.92E-03	1.56E+00
Cortical bone	2.99E-03	1.19E+00
ET region	2.13E-03	8.48E-01
ET1 surface	1.82E-03	7.24E-01
ET2 surface	2.13E-03	8.48E-01
Extrathoracic	4.27E-03	1.70E+00
Eye lenses	3.27E-03	1.30E+00
Gallbladder w	4.78E-03	1.90E+00
Heart wall	3.03E-03	1.20E+00
Kidneys	3.61E-03	1.44E+00
Left colon wa	3.78E-03	1.50E+00
Liver	3.88E-03	1.54E+00
Lungs	3.59E-03	1.43E+00
Lymphatic no	4.63E-03	1.84E+00
Muscle	1.11E-03	4.42E-01
Oesophagus	3.95E-03	1.57E+00
Oral mucosa	4.17E-03	1.66E+00
Ovaries	0.00E+00	0.00E+00
Pancreas	4.91E-03	1.95E+00
Pituitary glan	3.53E-03	1.40E+00
Prostate	5.95E-03	2.37E+00
Rectosigmoid	4.37E-03	1.74E+00
Red marrow	3.20E-03	1.27E+00
Right colon w	3.84E-03	1.53E+00
SI wall	3.98E-03	1.58E+00
Salivary glanc	3.93E-03	1.56E+00
Skin	3.46E-03	1.38E+00
Spleen	3.86E-03	1.54E+00
Stomach wall	3.64E-03	1.45E+00
Systemic lym	4.68E-03	1.86E+00
Testes	4.37E-03	1.74E+00
Thoracic lymp	4.53E-03	1.80E+00
Thymus	4.41E-03	1.75E+00
Thyroid	4.63E-03	1.84E+00
Tongue	2.52E-03	1.00E+00
Tonsils	4.06E-03	1.62E+00
Ureters	5.26E-03	2.09E+00

P4		
Male Adult Phantom		[MBq]
14.03.2019	14:27:04	388.12
**Organ**	**[mGy/MBq]**	**[mGy]**
Adipose	4.15E-03	1.61E+00
Adrenals	4.84E-03	1.88E+00
Alveolar-inte	4.09E-03	1.59E+00
Brain	1.14E-03	4.44E-01
Breasts	4.02E-03	1.56E+00
Bronchial bou	4.27E-03	1.66E+00
Bronchial seq	4.25E-03	1.65E+00
Bronchiolar s	4.19E-03	1.63E+00
Colon	3.87E-03	1.50E+00
Cortical bone	2.93E-03	1.14E+00
ET region	2.07E-03	8.03E-01
ET1 surface	1.76E-03	6.83E-01
ET2 surface	2.07E-03	8.03E-01
Extrathoracic	4.12E-03	1.60E+00
Eye lenses	3.15E-03	1.22E+00
Gallbladder w	5.34E-03	2.07E+00
Heart wall	2.67E-03	1.04E+00
Kidneys	4.09E-03	1.59E+00
Left colon wa	3.69E-03	1.43E+00
Liver	5.44E-03	2.11E+00
Lungs	4.18E-03	1.62E+00
Lymphatic no	4.55E-03	1.77E+00
Muscle	1.11E-03	4.29E-01
Oesophagus	3.93E-03	1.53E+00
Oral mucosa	4.02E-03	1.56E+00
Ovaries	0.00E+00	0.00E+00
Pancreas	5.01E-03	1.94E+00
Pituitary glan	3.44E-03	1.33E+00
Prostate	5.83E-03	2.26E+00
Rectosigmoid	4.27E-03	1.66E+00
Red marrow	3.21E-03	1.25E+00
Right colon w	3.84E-03	1.49E+00
SI wall	3.91E-03	1.52E+00
Salivary glanc	3.79E-03	1.47E+00
Skin	3.35E-03	1.30E+00
Spleen	3.81E-03	1.48E+00
Stomach wall	3.69E-03	1.43E+00
Systemic lym	4.60E-03	1.79E+00
Testes	4.20E-03	1.63E+00
Thoracic lymp	4.46E-03	1.73E+00
Thymus	4.33E-03	1.68E+00
Thyroid	3.99E-03	1.55E+00
Tongue	2.44E-03	9.45E-01
Tonsils	3.92E-03	1.52E+00
Ureters	5.14E-03	1.99E+00

Urinary bladc	8.82E-03	3.51E+00
Uterus/Cervi	0.00E+00	0.00E+00
	**[mSv/MBq]**	**[mSv]**
ED ICRP 103	4.10E-03	1.63E+00
ED ICRP 60	3.99E-03	1.59E+00

Urinary bladc	8.84E-03	3.43E+00
Uterus/Cervi	0.00E+00	0.00E+00
	**[mSv/MBq]**	**[mSv]**
ED ICRP 103	4.21E-03	1.64E+00
ED ICRP 60	4.07E-03	1.58E+00

P5		
Male Adult Phantom		[MBq]
21.03.2019	14:28:33	352.71
**Organ**	**[mGy/MBq]**	**[mGy]**
Adipose	4.08E-03	1.44E+00
Adrenals	5.00E-03	1.76E+00
Alveolar-inte	3.42E-03	1.21E+00
Brain	1.22E-03	4.31E-01
Breasts	3.93E-03	1.39E+00
Bronchial bou	4.00E-03	1.41E+00
Bronchial seq	3.99E-03	1.41E+00
Bronchiolar s	3.51E-03	1.24E+00
Colon	3.88E-03	1.37E+00
Cortical bone	2.89E-03	1.02E+00
ET region	2.02E-03	7.13E-01
ET1 surface	1.72E-03	6.05E-01
ET2 surface	2.02E-03	7.13E-01
Extrathoracic	4.01E-03	1.42E+00
Eye lenses	3.07E-03	1.08E+00
Gallbladder w	6.63E-03	2.34E+00
Heart wall	2.87E-03	1.01E+00
Kidneys	5.46E-03	1.92E+00
Left colon wa	3.64E-03	1.28E+00
Liver	5.98E-03	2.11E+00
Lungs	3.64E-03	1.28E+00
Lymphatic no	4.51E-03	1.59E+00
Muscle	1.11E-03	3.90E-01
Oesophagus	3.86E-03	1.36E+00
Oral mucosa	3.91E-03	1.38E+00
Ovaries	0.00E+00	0.00E+00
Pancreas	5.11E-03	1.80E+00
Pituitary glan	3.39E-03	1.19E+00
Prostate	6.13E-03	2.16E+00
Rectosigmoid	4.41E-03	1.55E+00
Red marrow	3.20E-03	1.13E+00
Right colon w	3.86E-03	1.36E+00
SI wall	3.93E-03	1.39E+00
Salivary glanc	3.69E-03	1.30E+00
Skin	3.27E-03	1.15E+00
Spleen	3.74E-03	1.32E+00
Stomach wall	3.67E-03	1.30E+00
Systemic lym	4.58E-03	1.61E+00
Testes	4.11E-03	1.45E+00
Thoracic lymp	4.32E-03	1.52E+00
Thymus	4.19E-03	1.48E+00
Thyroid	4.78E-03	1.69E+00
Tongue	2.37E-03	8.37E-01
Tonsils	3.83E-03	1.35E+00
Ureters	5.18E-03	1.83E+00

P6		
Male Adult Phantom		[MBq]
28.03.2019	14:30:00	353.39
**Organ**	**[mGy/MBq]**	**[mGy]**
Adipose	4.23E-03	1.49E+00
Adrenals	4.87E-03	1.72E+00
Alveolar-inte	4.11E-03	1.45E+00
Brain	8.96E-04	3.17E-01
Breasts	4.10E-03	1.45E+00
Bronchial bou	4.33E-03	1.53E+00
Bronchial seq	4.31E-03	1.52E+00
Bronchiolar s	4.20E-03	1.49E+00
Colon	3.88E-03	1.37E+00
Cortical bone	2.99E-03	1.06E+00
ET region	2.11E-03	7.44E-01
ET1 surface	1.80E-03	6.36E-01
ET2 surface	2.11E-03	7.44E-01
Extrathoracic	4.24E-03	1.50E+00
Eye lenses	3.23E-03	1.14E+00
Gallbladder w	5.16E-03	1.82E+00
Heart wall	2.61E-03	9.23E-01
Kidneys	4.24E-03	1.50E+00
Left colon wa	3.77E-03	1.33E+00
Liver	4.82E-03	1.70E+00
Lungs	4.21E-03	1.49E+00
Lymphatic no	4.59E-03	1.62E+00
Muscle	1.11E-03	3.91E-01
Oesophagus	4.00E-03	1.41E+00
Oral mucosa	4.12E-03	1.46E+00
Ovaries	0.00E+00	0.00E+00
Pancreas	5.03E-03	1.78E+00
Pituitary glan	3.43E-03	1.21E+00
Prostate	5.51E-03	1.95E+00
Rectosigmoid	4.12E-03	1.46E+00
Red marrow	3.27E-03	1.15E+00
Right colon w	3.87E-03	1.37E+00
SI wall	3.92E-03	1.38E+00
Salivary glanc	3.89E-03	1.37E+00
Skin	3.43E-03	1.21E+00
Spleen	3.90E-03	1.38E+00
Stomach wall	3.71E-03	1.31E+00
Systemic lym	4.63E-03	1.64E+00
Testes	4.29E-03	1.52E+00
Thoracic lymp	4.57E-03	1.61E+00
Thymus	4.44E-03	1.57E+00
Thyroid	4.55E-03	1.61E+00
Tongue	2.49E-03	8.81E-01
Tonsils	4.01E-03	1.42E+00
Ureters	5.15E-03	1.82E+00

Urinary bladc	1.02E-02	3.59E+00
Uterus/Cervi›	0.00E+00	0.00E+00
	**[mSv/MBq]**	**[mSv]**
ED ICRP 103	4.28E-03	1.51E+00
ED ICRP 60	4.11E-03	1.45E+00

Urinary bladc	7.40E-03	2.61E+00
Uterus/Cervi›	0.00E+00	0.00E+00
	**[mSv/MBq]**	**[mSv]**
ED ICRP 103	4.17E-03	1.47E+00
ED ICRP 60	4.03E-03	1.43E+00

P7		
Female Adult Phantom		[MBq]
11.04.2019	14:26:00	467.54
**Organ**	**[mGy/MBq]**	**[mGy]**
Adipose	4.18E-03	1.95E+00
Adrenals	5.03E-03	2.35E+00
Alveolar-inte	3.39E-03	1.58E+00
Brain	9.43E-04	4.41E-01
Breasts	4.13E-03	1.93E+00
Bronchial bou	4.17E-03	1.95E+00
Bronchial seq	4.16E-03	1.95E+00
Bronchiolar s	3.35E-03	1.57E+00
Colon	4.03E-03	1.89E+00
Cortical bone	2.83E-03	1.32E+00
ET region	2.33E-03	1.09E+00
ET1 surface	2.11E-03	9.88E-01
ET2 surface	2.33E-03	1.09E+00
Extrathoracic	4.37E-03	2.04E+00
Eye lenses	2.99E-03	1.40E+00
Gallbladder w	5.30E-03	2.48E+00
Heart wall	2.54E-03	1.19E+00
Kidneys	4.52E-03	2.11E+00
Left colon wa	3.86E-03	1.80E+00
Liver	6.12E-03	2.86E+00
Lungs	3.63E-03	1.70E+00
Lymphatic no	4.58E-03	2.14E+00
Muscle	1.37E-03	6.39E-01
Oesophagus w	3.73E-03	1.75E+00
Oral mucosa	3.89E-03	1.82E+00
Ovaries	5.85E-03	2.74E+00
Pancreas	4.79E-03	2.24E+00
Pituitary glan	3.51E-03	1.64E+00
Prostate	0.00E+00	0.00E+00
Rectosigmoid	4.94E-03	2.31E+00
Red marrow	3.20E-03	1.49E+00
Right colon w	3.75E-03	1.76E+00
SI wall	4.18E-03	1.96E+00
Salivary gland	3.97E-03	1.85E+00
Skin	3.37E-03	1.58E+00
Spleen	3.81E-03	1.78E+00
Stomach wall	3.68E-03	1.72E+00
Systemic lym	4.62E-03	2.16E+00
Testes	0.00E+00	0.00E+00
Thoracic lymp	4.39E-03	2.05E+00
Thymus	4.20E-03	1.96E+00
Thyroid	3.78E-03	1.77E+00
Tongue	2.18E-03	1.02E+00
Tonsils	3.91E-03	1.83E+00
Ureters	5.19E-03	2.42E+00

P8		
Female Adult Phantom		[MBq]
09.05.2019	14:24:00	418.96
**Organ**	**[mGy/MBq]**	**[mGy]**
Adipose	4.09E-03	1.71E+00
Adrenals	5.47E-03	2.29E+00
Alveolar-inte	3.89E-03	1.63E+00
Brain	9.79E-04	4.10E-01
Breasts	4.11E-03	1.72E+00
Bronchial bou	4.28E-03	1.79E+00
Bronchial seq	4.26E-03	1.79E+00
Bronchiolar s	3.85E-03	1.61E+00
Colon	3.92E-03	1.64E+00
Cortical bone	2.72E-03	1.14E+00
ET region	2.28E-03	9.54E-01
ET1 surface	2.07E-03	8.66E-01
ET2 surface	2.28E-03	9.54E-01
Extrathoracic	4.27E-03	1.79E+00
Eye lenses	2.92E-03	1.22E+00
Gallbladder w	6.24E-03	2.61E+00
Heart wall	2.74E-03	1.15E+00
Kidneys	5.52E-03	2.31E+00
Left colon wa	3.75E-03	1.57E+00
Liver	8.26E-03	3.46E+00
Lungs	4.00E-03	1.68E+00
Lymphatic no	4.53E-03	1.90E+00
Muscle	1.37E-03	5.74E-01
Oesophagus w	3.80E-03	1.59E+00
Oral mucosa	3.79E-03	1.59E+00
Ovaries	5.56E-03	2.33E+00
Pancreas	5.00E-03	2.09E+00
Pituitary glan	3.44E-03	1.44E+00
Prostate	0.00E+00	0.00E+00
Rectosigmoid	4.66E-03	1.95E+00
Red marrow	3.06E-03	1.28E+00
Right colon w	3.71E-03	1.55E+00
SI wall	4.10E-03	1.72E+00
Salivary gland	3.87E-03	1.62E+00
Skin	3.30E-03	1.38E+00
Spleen	3.85E-03	1.61E+00
Stomach wall	3.85E-03	1.61E+00
Systemic lym	4.57E-03	1.91E+00
Testes	0.00E+00	0.00E+00
Thoracic lymp	4.37E-03	1.83E+00
Thymus	4.16E-03	1.74E+00
Thyroid	3.33E-03	1.39E+00
Tongue	2.13E-03	8.92E-01
Tonsils	3.82E-03	1.60E+00
Ureters	5.08E-03	2.13E+00

Urinary bladd	1.13E-02	5.27E+00
Uterus/Cervix	6.84E-03	3.20E+00
	**[mSv/MBq]**	**[mSv]**
ED ICRP 103	4.18E-03	1.95E+00
ED ICRP 60	4.50E-03	2.11E+00

Urinary bladd	9.99E-03	4.19E+00
Uterus/Cervix	6.38E-03	2.67E+00
	**[mSv/MBq]**	**[mSv]**
ED ICRP 103	4.21E-03	1.77E+00
ED ICRP 60	4.50E-03	1.89E+00

P9		
Female Adult Phantom		[MBq]
16.05.2019	14:27:03	411.86
**Organ**	**[mGy/MBq]**	**[mGy]**
Adipose	4.03E-03	1.66E+00
Adrenals	5.05E-03	2.08E+00
Alveolar-inte	3.48E-03	1.43E+00
Brain	9.61E-04	3.96E-01
Breasts	3.97E-03	1.63E+00
Bronchial bou	4.07E-03	1.68E+00
Bronchial seq	4.06E-03	1.67E+00
Bronchiolar s	3.44E-03	1.42E+00
Colon	4.09E-03	1.69E+00
Cortical bone	2.73E-03	1.12E+00
ET region	2.22E-03	9.13E-01
ET1 surface	2.01E-03	8.28E-01
ET2 surface	2.22E-03	9.14E-01
Extrathoracic	4.15E-03	1.71E+00
Eye lenses	2.84E-03	1.17E+00
Gallbladder w	5.49E-03	2.26E+00
Heart wall	2.66E-03	1.09E+00
Kidneys	4.71E-03	1.94E+00
Left colon wa	3.83E-03	1.58E+00
Liver	6.95E-03	2.86E+00
Lungs	3.66E-03	1.51E+00
Lymphatic no	4.51E-03	1.86E+00
Muscle	1.37E-03	5.65E-01
Oesophagus	3.63E-03	1.50E+00
Oral mucosa	3.69E-03	1.52E+00
Ovaries	6.20E-03	2.55E+00
Pancreas	4.72E-03	1.94E+00
Pituitary glan	3.35E-03	1.38E+00
Prostate	0.00E+00	0.00E+00
Rectosigmoid	5.39E-03	2.22E+00
Red marrow	3.11E-03	1.28E+00
Right colon w	3.71E-03	1.53E+00
SI wall	4.32E-03	1.78E+00
Salivary glan	3.77E-03	1.55E+00
Skin	3.24E-03	1.33E+00
Spleen	3.68E-03	1.52E+00
Stomach wall	3.63E-03	1.50E+00
Systemic lym	4.57E-03	1.88E+00
Testes	0.00E+00	0.00E+00
Thoracic lym	4.23E-03	1.74E+00
Thymus	4.02E-03	1.66E+00
Thyroid	4.08E-03	1.68E+00
Tongue	2.07E-03	8.53E-01
Tonsils	3.72E-03	1.53E+00
Ureters	5.25E-03	2.16E+00

P10		
Female Adult Phantom		[MBq]
14.11.2019	14:36:00	422.21
**Organ**	**[mGy/MBq]**	**[mGy]**
Adipose	4.22E-03	1.78E+00
Adrenals	5.12E-03	2.16E+00
Alveolar-inte	3.99E-03	1.69E+00
Brain	9.85E-04	4.16E-01
Breasts	4.23E-03	1.79E+00
Bronchial bou	4.43E-03	1.87E+00
Bronchial seq	4.42E-03	1.87E+00
Bronchiolar s	3.96E-03	1.67E+00
Colon	3.94E-03	1.66E+00
Cortical bone	2.78E-03	1.17E+00
ET region	2.37E-03	9.99E-01
ET1 surface	2.15E-03	9.07E-01
ET2 surface	2.37E-03	9.99E-01
Extrathoracic	4.44E-03	1.87E+00
Eye lenses	3.04E-03	1.28E+00
Gallbladder w	5.62E-03	2.37E+00
Heart wall	3.12E-03	1.32E+00
Kidneys	4.04E-03	1.70E+00
Left colon wa	3.82E-03	1.61E+00
Liver	6.46E-03	2.73E+00
Lungs	4.13E-03	1.74E+00
Lymphatic no	4.60E-03	1.94E+00
Muscle	1.36E-03	5.76E-01
Oesophagus	3.86E-03	1.63E+00
Oral mucosa	3.95E-03	1.67E+00
Ovaries	5.56E-03	2.35E+00
Pancreas	4.87E-03	2.06E+00
Pituitary glan	3.57E-03	1.51E+00
Prostate	0.00E+00	0.00E+00
Rectosigmoid	4.60E-03	1.94E+00
Red marrow	3.06E-03	1.29E+00
Right colon w	3.74E-03	1.58E+00
SI wall	4.06E-03	1.71E+00
Salivary glan	4.03E-03	1.70E+00
Skin	3.41E-03	1.44E+00
Spleen	3.88E-03	1.64E+00
Stomach wall	3.76E-03	1.59E+00
Systemic lym	4.62E-03	1.95E+00
Testes	0.00E+00	0.00E+00
Thoracic lym	4.54E-03	1.92E+00
Thymus	4.32E-03	1.82E+00
Thyroid	3.43E-03	1.45E+00
Tongue	2.21E-03	9.34E-01
Tonsils	3.98E-03	1.68E+00
Ureters	5.07E-03	2.14E+00

Urinary bladc	1.51E-02	6.21E+00
Uterus/Cervi›	7.77E-03	3.20E+00
	**[mSv/MBq]**	**[mSv]**
ED ICRP 103	4.33E-03	1.78E+00
ED ICRP 60	4.80E-03	1.98E+00

Urinary bladc	8.87E-03	3.75E+00
Uterus/Cervi›	6.19E-03	2.61E+00
	**[mSv/MBq]**	**[mSv]**
ED ICRP 103	4.13E-03	1.75E+00
ED ICRP 60	4.38E-03	1.85E+00

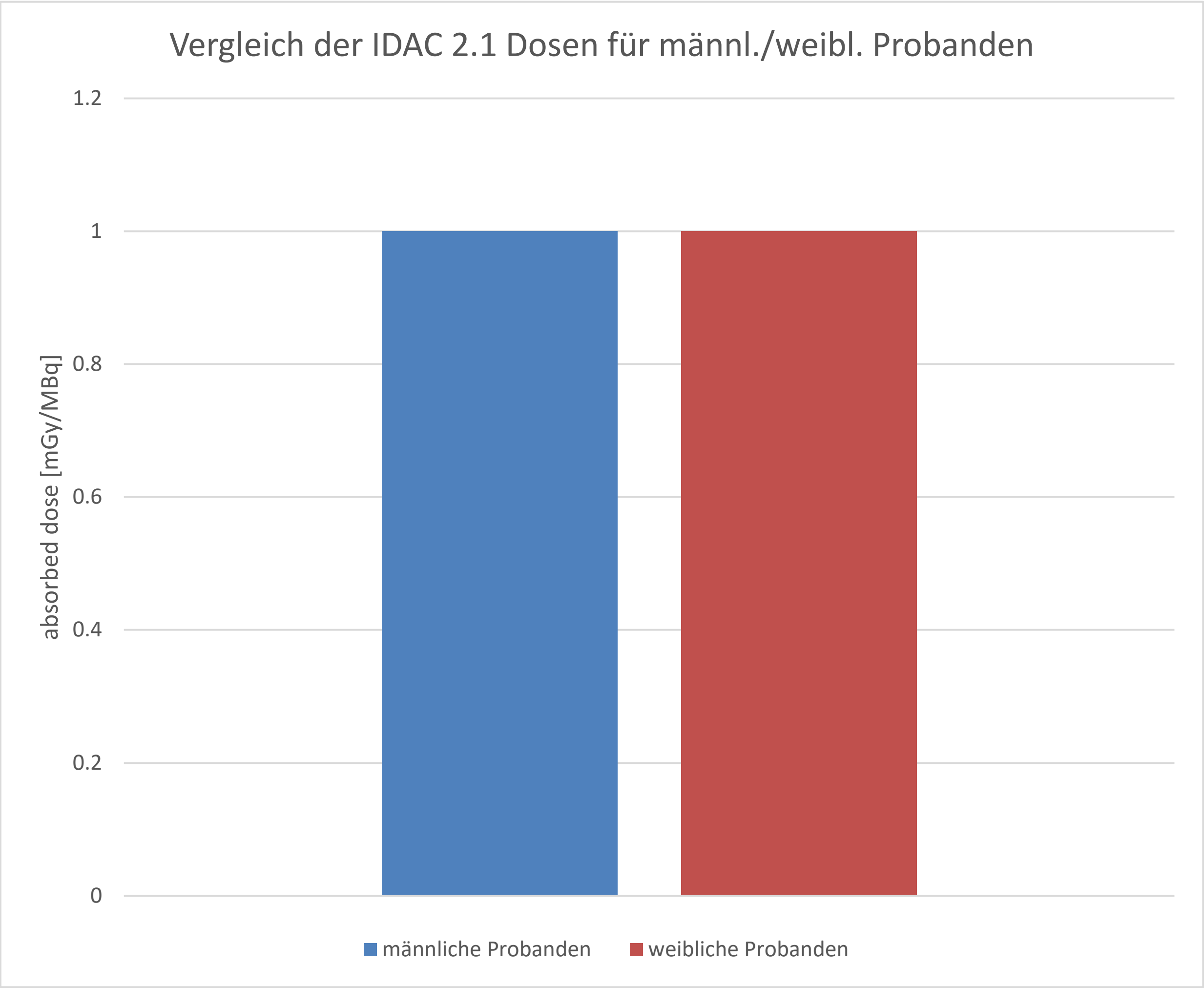
Vergleich der IDAC 2.1 Dosen für männl./weibl. Probanden
1.2
1
0.8
0.6
0.4
0.2
0
absorbed dose [mGy/MBq]
männliche Probanden
weibliche Probanden
